# Baseline Insulin Resistance Is a Determinant of the Small, Dense Low-Density Lipoprotein Response to Diets Differing in Saturated Fat, Protein, and Carbohydrate Contents

**DOI:** 10.3390/nu13124328

**Published:** 2021-11-30

**Authors:** Xiuzhi Wu, Michael A. Roussell, Alison M. Hill, Penny M. Kris-Etherton, Rosemary L. Walzem

**Affiliations:** 1 Department of Nutrition and Food Science, Texas A&M University, College Station, TX 77843, USA; xiuzhiwu@tamu.edu; 2 Department of Nutritional Sciences, Pennsylvania State University, University Park, State College, PA 16802, USA; mike@mikerousell.com (M.A.R.); alison.hill@unisa.edu.au (A.M.H.); pmk3@psu.edu (P.M.K.-E.); 3 UniSA Clinical & Health Sciences, University of South Australia, Adelaide, SA 5000, Australia; 4 Department of Poultry Science, Texas A&M University, College Station, TX 77843, USA; 5 Faculty of Nutrition, Texas A&M University, College Station, TX 77843, USA

**Keywords:** insulin resistance, diet response, beef consumption, saturated fat

## Abstract

Individual responses to diet vary but causes other than genetics are poorly understood. This study sought to determine whether baseline values of homeostasis model assessment (HOMA-IR) was related to changes in small, dense low-density lipoprotein (sdLDL, i.e., LDL_4_, d = 1.044–1.063 g/mL) amounts quantified by isopycnic density profiling, in mildly hypercholesterolemic subjects (*n* = 27) consuming one of three low saturated fatty acid (SFA) diets: Dietary Approaches to Stop Hypertension (DASH), Beef in an Optimal Lean Diet (BOLD) and BOLD plus extra protein (BOLD+) when compared to a higher-SFA healthy American diet (HAD). The diets were consumed in random order for 5 wk, with 1 wk between diets. BOLD+ reduced fractional abundance (%) LDL_4_ (*p* < 0.05) relative to HAD, DASH and BOLD, and reductions in % LDL_4_ correlated with reductions in triglycerides (*p* = 0.044), total cholesterol (*p* = 0.014), LDL cholesterol (*p* = 0.004) and apolipoprotein B (*p* < 0.001). Responses to the four diets were similar (~12% decrease in % LDL_4_, *p* = 0.890) in the lower (<2.73 median) HOMA-IR subgroup but differed across diet conditions in the higher HOMA-IR subgroup (*p* = 0.013), in which % LDL_4_ was reduced with BOLD+ (−11%), was unchanged in BOLD and increased with the HAD (8%) and DASH (6%) diets (*p* < 0.05 for BOLD+ vs. HAD). Individual responses to diet interventions are influenced by presence and degree of insulin resistance as measured by HOMA-IR.

## 1. Introduction

Low-density lipoprotein (LDL) particles are highly heterogeneous, and likely vary in atherogenicity due to differences in size, composition, and physiochemical properties. Compared with larger LDL particles, small dense LDL (sdLDL) particles have a higher propensity to penetrate the artery wall and bind to proteoglycans [1], increased susceptibility to oxidation [2] and reduced affinity for the LDL receptor [3]. Thus, a higher level of sdLDL particles is associated with increased risk for ischemic heart disease [4].

Lifestyle change to alter modifiable risk factors is often the first strategy to reduce ischemic heart disease, with reductions in total and LDL cholesterol (LDL-C) being key targets for reduction. Intervention studies demonstrate that LDL density distribution is modulated by dietary macronutrient composition. Compared with an isocaloric high-fat diet, low fat/high carbohydrate diets are associated with either no change or a decrease in LDL particle diameter (i.e., a shift toward sdLDL) [5,6,7,8,9]. The differences among studies were ascribed, in part, to differences in type (simple or complex) and proportion of energy consumed from carbohydrate (54–75% of total energy). Replacement of carbohydrate by protein (from plant or animal sources) in moderate- or high-fat eucaloric diets is generally associated with an improved LDL density profile, with a shift away from sdLDL toward larger, more buoyant LDL subfractions [8,10,11].

LDL particle diameter is related to LDL density [10]. Considerable within-study variation in individual LDL particle diameter responsiveness to diet has been consistently observed, even under controlled feeding conditions, suggesting an influence of genetic and other environmental factors. For example, apolipoprotein E phenotype [5] and sdLDL subclass pattern B [9] both have been identified as determinants of LDL responses to lower fat diets. Others have proposed that a tipping point exists where insulin resistance becomes inflammatory insulin resistance [11], and Gower et al. [12] reported that improved insulin sensitivity in response to 30 g of resistant starch differed depending on the subject’s baseline insulin sensitivity. Further, the presence of sdLDL, or indirect indices of sdLDL, were associated with insulin resistance and diabetes in a large cross-sectional study in China [13], and with pregnancy complications in nonobese women with polycystic ovary syndrome [14].

We could find no publications reporting the effects of baseline insulin sensitivity, as judged by homeostasis model assessment (HOMA-IR), on lipoprotein density distribution response to isocaloric diets that were lower in total and saturated fatty acids (SFA) that varied in carbohydrate or protein replacement calories in subjects whose triacylglycerol, insulin and glucose values were within normal limits [15,16].

The Dietary Approaches to Stop Hypertension (DASH) dietary pattern is a recommended heart healthy diet that emphasizes fruits and vegetables and is low in SFA and cholesterol [17,18]. Some epidemiological studies reported adverse health associations for red meat intake, which may be attributable to its SFA content, varying levels of meat processing and additives, residual confounding, or other factors [19,20,21]. Studies using fresh beef in interventions did not reliably show elevations in cardiovascular risk factors [11,22,23]. The Beef in an Optimal Lean Diet (BOLD) study compared the effects of a DASH dietary pattern with two DASH-like dietary patterns that provided different amounts of lean beef in a mildly hypercholesterolemic study sample to assess the effects of lean beef on cardiometabolic health markers in the context of healthy diet patterns [22]. The primary findings, reported previously, were the significant (and similar) reductions in total and LDL cholesterol levels in the DASH and lean beef-containing (BOLD and BOLD+) diets, compared to the healthy American control diet (HAD) [22]. In addition, the higher protein/higher lean beef BOLD+ diet was the only dietary intervention to elicit a significant reduction in systolic blood pressure compared to the control diet [23]. Samples from the BOLD study provided the opportunity to examine the effect of replacing fat calories with protein or carbohydrate calories in a well-controlled dietary setting with mildly hypercholesterolemic, but otherwise healthy subjects [22]. While the BOLD study made chemical measurements of fasting plasma lipids, it did not assess whether cholesterol reductions were associated with reductions in sdLDL. Thus, the primary aim of this secondary analysis was to assess the effects of dietary patterns that varied in the amount of protein provided by lean beef on lipoprotein density distributions, with an emphasis on changes in the abundance of sdLDL. In addition, inflammatory status and insulin sensitivity have been associated with changes in lipoprotein particle diameter in prior studies [24]. Therefore, secondary aims were to assess whether inflammatory status and insulin sensitivity at baseline were related to lipoprotein density distribution response to the different diets.

## 2. Materials and Methods

### 2.1. Participants and Design

The present study is a secondary analysis, conducted in 2014, using all 27 of the complete serum sample sets remaining from the original BOLD study [22]. Lack of a complete sample set was the sole exclusion criteria for the secondary analysis. The experimental design and measurements of the original study were described in detail previously [22]. Briefly, a 4-period, randomized, cross-over, controlled-feeding design was used. Participants consumed each of four diets: HAD, DASH, BOLD and BOLD+ for 5 weeks in a random order in a controlled-feeding setting with a minimum of a 1-wk compliance break between the diets. The macronutrient and fatty acid distributions of the four test diets are shown in Table 1. At the beginning of the study and at the end of each diet period, serum samples were taken from healthy men (*n* = 10) and women (*n* = 17) (36–65 years of age) with moderately high baseline LDL-C (>2.8 mmol/L). Participants were not on any lipid-lowering medications or dietary supplements during the study. Insulin resistance was not a factor in subject selection. Serum glucose and insulin concentrations were available for 25 of the 27 subjects.

The HAD was higher in SFA and total fat than the other three test diets. It also included more refined grains, added sugars, and full-fat dairy products compared to the test diets. The DASH, BOLD and BOLD+ diets included more whole grains, vegetables, fruits, and low-fat dairy products. The DASH and BOLD diets were matched for macronutrient energy distribution, but differed in the amounts of beef included, 28 g/d and 113 g/d lean beef respectively. The BOLD+ diet contained similar proportions of total and saturated fat as the BOLD and DASH diets but contained less carbohydrate (45% vs. 54–55% energy, respectively) and greater amounts of lean beef (153 g/d) and total protein (27% vs. 18–19% energy, respectively). The total energy content of the test diets was individually adjusted to keep participants’ weight stable during the 4 feeding periods. In the original study, participants consumed one meal per weekday in the Penn State Metabolic Diet Study Center, with the other meals being prepared and packed for off-site consumption. Diet adherence was monitored by daily and weekly compliance questionnaires.

### 2.2. Laboratory Assessment

Coded samples were received from the Penn State laboratory and analyzed for lipoprotein density distribution prior to revelation of treatment codes and statistical analysis. Analytical methods for clinical chemistry and immunoassays were published in detail previously [22]. Briefly, lipid concentrations were measured enzymatically at the MS Hershey Medical Center General Clinical Research Center (Hershey, Derry, PA, USA); this same core facility measured insulin by radioimmunoassay using ^125^I-labeled human insulin and human insulin antiserum [25] and glucose by an immobilized enzyme biosensor for glucose [26]. Apolipoprotein B was measured by immunoturbidimetric assay at the Oklahoma Research Institute (Oklahoma City, OK, USA) under the supervision of Petar Alaupovic. High-sensitivity CRP (hsCRP) was measured with the use of latex-enhanced immunonephelometric assay (Quest Diagnostics, NJ, USA).

Insulin resistance (inversely related to insulin sensitivity) was estimated by HOMA-IR [27] calculated from previously measured serum glucose and insulin concentrations using the following equation:

$$\mathrm{HOMA}-\mathrm{IR}=\frac{\mathrm{Glucose}\;\times \;\mathrm{Insulin}}{22.5}$$

Glucose and insulin are expressed in mmol/L and mU/L, respectively.

Lipoprotein density profile was determined by imaging of fluorescently stained lipoproteins following NaBiEDTA ultracentrifugation as previously described with modifications [28]. Briefly, 6 µL serum was incubated with 10 µL 1 g/L 6-((N-(7-Nitrobenz-2-Oxa-1,3-Diazol-4-yl)amino)hexanoyl) Sphingosine (i.e. NBD-C6-ceramide), and 1184 µL 0.18 mol/L NaBiEDTA density gradient solution. This solution (1150 µL) was then transferred to an 11 mm × 34 mm thickwall polycarbonate tube and centrifuged in a Beckman Optima MAX-XP centrifuge equipped with an MLA-130 rotor (Beckman Coulter, Inc. Brea, CA, USA) for 6 hr at 4 °C. Following gentle overlayment of hexane, the tubes were immediately imaged by a CCD camera (Quantifire XI, Optronics, Muskogee, OK, USA) with a Fiber-Lite Illuminator (MH100A, Edmund Industrial Optics, Barrington, NJ, USA) as light source. The tube holder, digital camera and illuminator were positioned orthogonally to each other on an optical bench. The respective filters (Semrock, Rochester, NY, USA) were chosen to match NBD excitation (465 nm/60 nm bandwidth, part # FF01-460-60-25) and emission (> 500 nm/long pass, part # BLP01-488R-25) wavelengths, respectively. Pixel values of the center of the tube were converted into fluorescent intensity using Origin 7.5 (OriginLab Ltd., Northampton, MA, USA) software and plotted as a function of the tube coordinate. A total of 10 lipoprotein subclasses were identified by their density intervals [29,30], and quantified by calculation of the area under the curve (AUC), i.e., pixel value. The major lipoprotein subclasses were triacylglycerol-rich lipoproteins (TRL; d < 1.019 g/mL), LDL_1_ (d = 1.019–1.023 g/mL), LDL_2_ (d = 1.023–1.034 g/mL), LDL_3_ (d = 1.034–1.044 g/mL), LDL_4_ (d = 1.044–1.063 g/mL), HDL_2b_ (d = 1.063–1.091 g/mL), HDL_2a_ (d = 1.091–1.110 g/mL), HDL_3a_ (d = 1.110–1.133 g/mL), HDL_3b_ (d = 1.133–1.156 g/mL) and HDL_3c_ (d = 1.156–1.179 g/mL) [31]. NBD-C6-ceramide only fluoresces in a hydrophobic environment. Thus, the fluorescent intensity of a particle depends on the quantity of the hydrophobic group/molecules in lipoprotein particles and should correlate to chemical determinations of lipids. Isopycnic density profiling used here defines sdLDL as LDL_4_ (d = 1.044–1.063 g/mL). The density profiling methodology was validated by measuring intra- and inter- assay reproducibility of samples differing in triacylglycerol (TG) concentration. Participants were grouped into quintiles based on serum TG concentration. Three serum samples from each of the 1st, 3rd, 5th quintiles were randomly selected and pooled and used as low (0.63 mmol/L), medium (1.15 mmol/L) and high (2.25 mmol/L) TG concentration samples. For intra-assay variation assessment, 3 replicates for each of the low, medium, and high TG samples were analyzed in a single run. For inter-assay variation assessment, 5 replicates of the samples were run on 5 separate days. On each day, a set of low, medium, and high TG samples was analyzed.

### 2.3. Statistics

Statistical analysis was performed using JMP 10.0 (SAS Institute Inc., Cary, NC, USA). Distributions were assessed for normality using the Shapiro-Wilk test. Continuous data with a skewed distribution were log transformed before analysis. Baseline characteristics between men and women were compared using two-sample t-tests. Further measurement of lipoprotein subclass by NBD-C6 ceramide distribution was compared with conventional chemical measurements from Roussell et al. [22] (see Table 2 and Table 3 of this report for those chemical values) using Pearson correlation. Forest plots were determined to visualize data prior to further statistical analysis (Appendix A Figure A2). The lipoprotein density distribution and HOMA-IR at baseline and after each of the four test diets were compared by repeated measures analysis of covariance with adjustment for age, gender, and BMI, followed by Tukey-Kramer test. The primary outcomes were the differences in absolute and fractional abundance of LDL_4_ after the consumption of the BOLD+ diet compared to the HAD diet. Pearson correlation coefficients were calculated for the BOLD+ diet induced change in LDL_4_ and changes in previously reported lipid variables and apolipoprotein concentrations. To examine the effects of baseline lipids, inflammation indicators and HOMA-IR on LDL_4_ response to test diets, Pearson correlation coefficients were calculated for baseline TG, total cholesterol (TC), LDL-C, HDL cholesterol (HDL-C), HOMA-IR, and CRP with percentage change of LDL_4_ from baseline for each test diet. When a significant correlation was observed for baseline HOMA-IR and LDL_4_ percentage change, a secondary analysis was performed to better characterize the effect of baseline HOMA-IR. Participants with baseline HOMA-IR below the median were classified as a lower HOMA-IR subgroup (*n* = 13) whereas participants with HOMA-IR equal or greater than the median were classified as a higher HOMA-IR subgroup (*n* = 12). To investigate the responsiveness to test diets within each baseline HOMA-IR subgroup, mixed model repeated measures analysis of covariance was performed with participants entered as a random variable and age, gender, and BMI as covariates. When a significant overall dietary effect was detected by the mixed model, the Tukey-Kramer test was used to further investigate the pairwise differences between diets. The level of significance was *p* < 0.05. Values for continuous variables were expressed as mean ± SEM.

## 3. Results

### 3.1. Baseline Characteristics

Baseline characteristics of the 27 participants in the original BOLD study [22] for whom complete sample sets were available are shown in Table 2. The participants were mildly hypercholesterolemic but were within the normal range for TG (<1.7 mmol/L) and HDL-C (>1.04 mmol/L for men and >1.30 mmol/L for women). Baseline serum glucose and insulin concentrations did not exceed normal limits [15,16].

**Figure A1:**
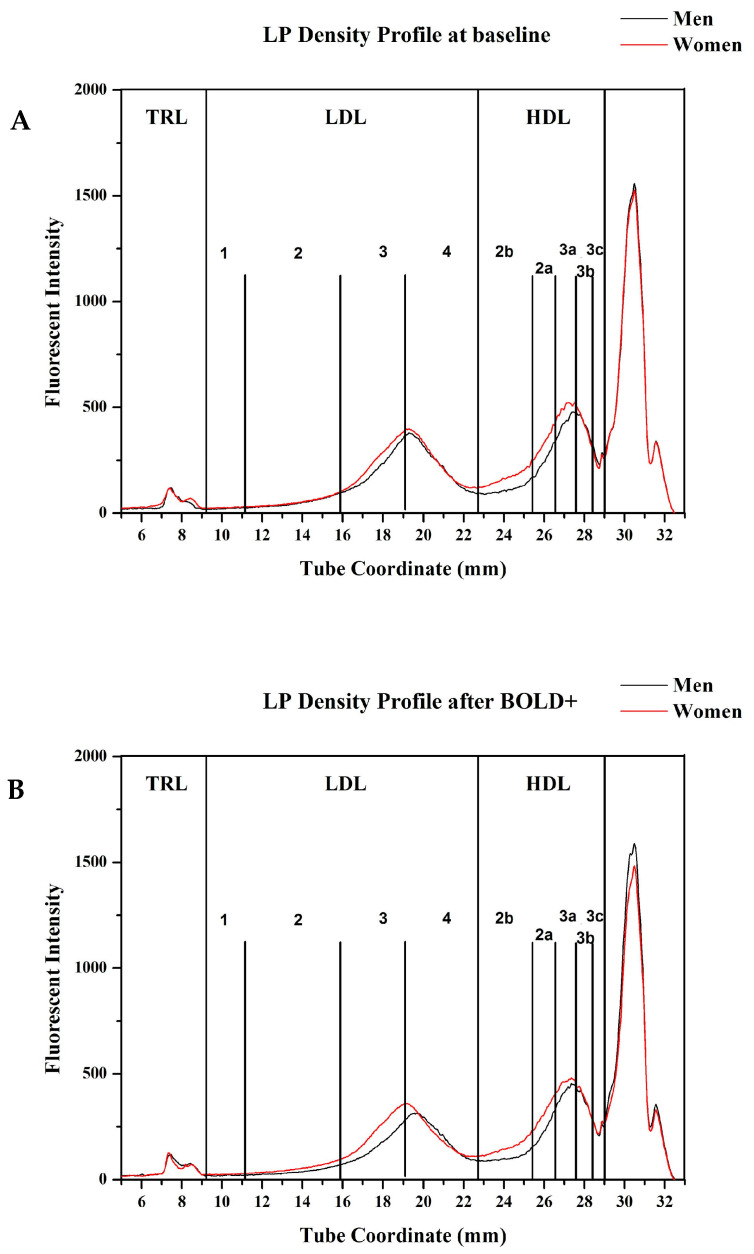
Lipoprotein density profile for male and female participants at baseline (**A**) and after dietary intervention (**B**). The BOLD+ diet was used as an example. No significant difference was detected for a gender effect on LDL_4_ distribution.

**Figure A2:**
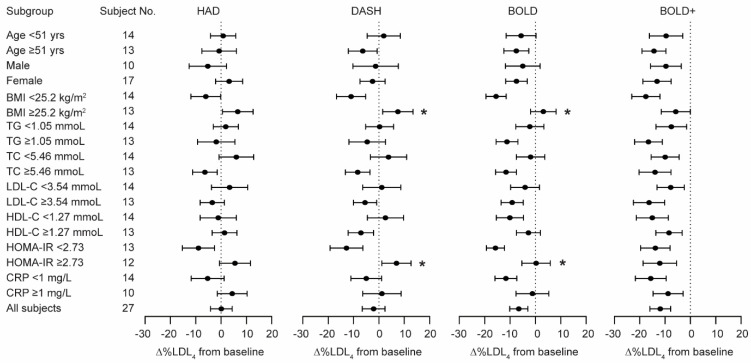
Forest plot. Subgroup analysis for LDL_4_ response after the 4 test diets. Data are mean ± SEM. * *p* < 0.05 between subgroup categories within each diet group as assessed using *t* test. Skewed data were log transformed before statistical analysis.

Females had a significantly higher average TC (*p* = 0.003) and LDL-C (*p* = 0.007) than males. No differences were observed for TG, HDL-C, CRP and HOMA-IR between men and women. Despite the difference in total LDL-C concentration between men and women, the difference was mainly due to the presence of more large and medium LDL, but not LDL_4_, in women (Appendix A Figure A1). No significant gender effect on LDL_4_ response to the test diets was observed. Baseline HOMA-IR values varied from 1.27 to 4.73 with a median of 2.73.

### 3.2. Post-Diet Characteristics

Table 3 summarizes previously reported [22] clinical and biochemical parameters used in lipoprotein density distribution correlation analyses. Mean values for BMI, TG, apolipoprotein B (ApoB), HOMA-IR and CRP were similar across dietary treatments. TC was reduced compared to baseline by DASH, BOLD and BOLD+; only BOLD+ reduced TC compared to HAD. Values for LDL-C were reduced by DASH, BOLD and BOLD+ when compared to baseline; no differences were observed compared to HAD. All diets lower in total and SFA reduced HDL-C compared to baseline, and both DASH and BOLD+ reduced HDL-C compared to HAD.

### 3.3. Lipoprotein Density Distribution

**Figure 1:**
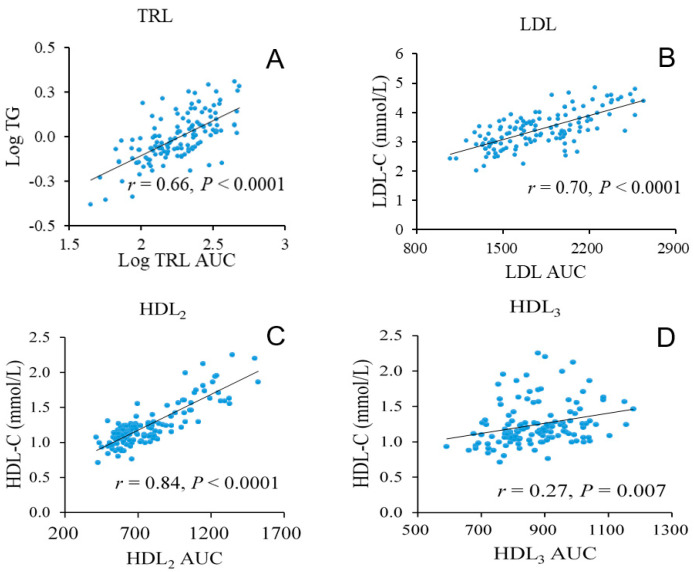
Correlations between lipoprotein density profile and biochemical measurements in 135 samples. Correlations are shown for TRL AUC with TG (**A**), LDL AUC with LDL-C (**B**), HDL_2_ AUC with HDL-C (**C**), HDL_3_ AUC with HDL-C (**D**). Linear regression, correlation coefficient and *p* value are shown for each comparison. TG and TRL AUC data were log transformed to achieve normality.

The AUC of core-lipid rich TRL, LDL and HDL_2_ were strongly correlated with chemically determined values for serum TG, LDL-C and HDL-C respectively (*r* ≥ 0.66, *p* < 0.0001 for all, Figure 1A–C). For cholesterol-poor HDL_3_, there was a significant, but weaker association with HDL-C (*r* = 0.27, *p* = 0.007, Figure 1D). The lipoprotein density distribution demonstrated that technical variations across different lipoprotein subclasses were similar for samples containing differing amounts of TG, with the highest variation occurring at the top and bottom of the regions of the tube (i.e., TRL, LDL_1,_ and HDL_3c_ subclasses). The increase in variability was likely due to the specific solution redistribution condition at these positions during centrifuge deceleration. For other lipoprotein subclasses, the average coefficient of variation (CV) was as low as 5% with 3 replicates for intra-assay assessments and 4% with 5 replicates for inter-assay assessments (Appendix A Table A1). Compared with baseline, no absolute or fractional change was observed for any lipoprotein subclass after the consumption of the HAD diet (Table 4). These observations support the conclusion that the macronutrient distribution of the participants’ baseline diet was similar to HAD.

While the lack of differences in lipoprotein density distributions observed during baseline and HAD periods lead to the conclusion that the macronutrient compositions of baseline and HAD were similar, statistical comparisons were made relative to outcomes from the HAD controlled feeding period. Compared with the control, HAD diet, BOLD+ significantly reduced total LDL_4_ AUC by ~9.2% which also caused a reduction in the fractional abundance (%) of LDL_4_ (*p* < 0.05), indicating a shift in LDL particle diameter distribution towards larger sizes. Further analysis demonstrated that when participants consumed the BOLD+ dietary pattern, the induced decreases in %LDL_4_ paralleled the decreases in TC (*r* = 0.48, *p* = 0.014), LDL-C (*r* = 0.55, *p* = 0.004) and ApoB (*r* = 0.68, *p* < 0.001), with the strongest association observed for ApoB. Despite the lack of a significant difference in mean TG after the consumption of the HAD vs. the BOLD+ diet, we observed a positive correlation between the change in TG and LDL_4_ (*r* = 0.41, *p* = 0.044). The BOLD+ diet did not result in decreases in the AUC of larger diameter LDL_2_ and LDL_3_ particles, while both DASH and BOLD decreased the AUC of these LDL subclasses by an average of ~4.6% and ~8.8%, respectively. Total HDL_2b_ AUC was significantly decreased by ~5.6% and ~6.8% after the consumption of the DASH and BOLD diets, respectively (*p* < 0.05), but not after consumption of the BOLD+ diet. The fractional abundance of HDL_3a_ increased following consumption of all three test diets low in SFA (i.e., DASH, BOLD and BOLD+) compared to the proportion present following consumption of the HAD (*p* < 0.05).

### 3.4. LDL_4_ Responsiveness

To identify possible predictors for individual LDL subclass responses to the HAD, DASH, BOLD and BOLD+ diets, univariate correlations were calculated for the percentage change in LDL_4_ from baseline with baseline lipid variables, HOMA-IR, and CRP. The results showed that increased baseline HOMA-IR was associated with a less favorable LDL_4_ response to the HAD (*r* = 0.36, *p* = 0.065), DASH (*r* = 0.56, *p* = 0.003) and BOLD (*r* = 0.47, *p* = 0.018) diets (Table 5). No such clear relationship existed for baseline HOMA-IR with LDL_4_ response to BOLD+ (*r* = 0.13, *p* = 0.533).

**Figure 2:**
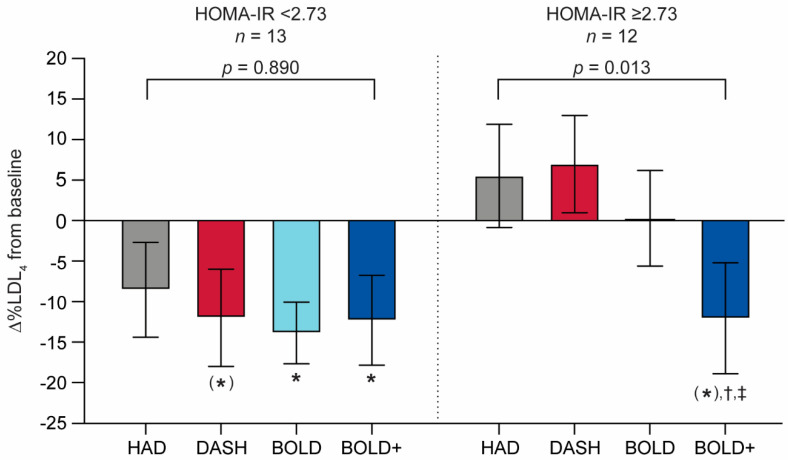
Effects of baseline HOMA-IR on LDL_4_ response. Lower and higher baseline HOMA-IR subgroups were classified by median split. Values were expressed as mean ± SEM. Within each subgroup, %LDL_4_ changes from baseline were compared by repeated measures analysis of covariance with adjustment for age, gender, and BMI. * *p* < 0.05 vs. zero; (*) *p* < 0.1 vs. zero. Difference in direction of change within HOMA-IR split group, ^†^ *p* < 0.05 vs. HAD; ^‡^ *p* < 0.05 vs. DASH (Tukey HSD). HAD = healthy American diet; DASH = Dietary Approaches to Stop Hypertension; BOLD = Beef in an Optimal Lean Diet; BOLD+ = BOLD plus extra protein.

To further assess the effect of baseline HOMA-IR on the LDL_4_ response, participants were grouped into lower and higher baseline HOMA-IR subgroups by median split (< or ≥2.73). Baseline subject characteristics following regrouping by median split are shown in Appendix A Table A2. The responses for %LDL_4_ for these two groups are shown in Figure 2. The responsiveness to the four diets, expressed as the change in %LDL_4_ from baseline, showed no significant difference (*p* = 0.890) in direction among participants with baseline HOMA-IR values ≤ 2.73; and all showed reductions in %LDL_4_. However, within this subgroup, as indicated by unbracketed asterisks, comparison of baseline to individual test diet endpoints demonstrated a significant decrease in %LDL_4_ after the consumption of the BOLD (*p* = 0.003) and BOLD+ (*p* = 0.046) diets and no significant decrease after the consumption of the DASH (*p* = 0.069) or HAD (*p* > 0.1) diets. In contrast, directionality of responses to the test diets differed among participants with baseline HOMA-IR > 2.73. The %LDL_4_ decreased in response to the BOLD+ diet, a direction that was significantly different from those for the HAD and DASH diets in the high HOMA-IR group (*p* = 0.013). Indeed, following the consumption of BOLD+ diet, %LDL_4_ was numerically lowered in the higher HOMA-IR subgroup (*p* < 0.1), with a magnitude comparable to that observed with all of the diets among subjects with lower HOMA-IR. However, among subjects with higher HOMA-IR values, no trend of decrease in %LDL_4_ from baseline was observed following the consumption of other three test diets.

## 4. Discussion

The participants in the original BOLD study, while being mildly hypercholesterolemic, were normotensive [23] with serum concentrations of TG, glucose, insulin, and HDL-C within normal limits [15,16,24] (and Table 2). Further, subjects maintained their starting bodyweights within 2.2 kg [22] while consuming the four isocaloric test diets over the >23 wk experimental period. Despite the lack of overt evidence of insulin resistance or metabolic syndrome, the ability of three different diets low in total and SFA to reduce the amounts of sdLDL varied in relation with HOMA-IR. Specifically, we found that increasing dietary protein, mainly from 153 g/day of lean beef in the BOLD + diet, while decreasing carbohydrate within a DASH-like dietary pattern improved the lipoprotein density profile by decreasing the absolute and proportional abundance of sdLDL, (i.e., LDL_4_) compared to a HAD diet. The degree of improvement was significant in individuals with HOMA-IR values ≤ 2.73; with BOLD+, the difference versus HAD was significant regardless of baseline HOMA-IR. The DASH and BOLD diets also appeared to have beneficial effects on the LDL density profile for some individuals, albeit improvements were less than those observed following the BOLD+ diet. The effects of these two diets appeared to also be influenced by baseline HOMA-IR value. To the best of our knowledge, this is the first study to show that baseline HOMA-IR provides predictive information regarding LDL_4_ response to a low SFA dietary intervention in normoglycemic individuals.

In the original report from the BOLD study, consumption of the BOLD+ diet reduced LDL cholesterol by 6% compared to HAD. We now show that cholesterol decrement was associated with a 9.2% decrease in the sdLDL (i.e., LDL_4_) species that is thought to be the most atherogenic, consistent with a shift toward a less atherogenic distribution of LDL particles [32,33].

In this analysis, LDL_4_ particles were defined as those with a density range of 1.044–1.063 g/mL, which roughly corresponds to LDL particles with a diameter smaller than 24.2 nm as measured by non-denaturing gradient gel electrophoresis [29]. The prospective Quebec Cardiovascular Study (*n* = 2034 men) found that participants whose absolute or relative cholesterol concentration in LDL particles with a diameter smaller than 25.5 nm had the strongest association with the risk of ischemic heart disease (relative risk = 4.6 in men in the third vs. first tertile of the distribution, *p* < 0.001), after adjustments for other lipid and non-lipid risk factors [34]. These observations suggest that the 9.2% decrease in sdLDL on the BOLD+ diet might elicit a clinically relevant benefit for cardiovascular disease risk.

Our finding that consistent directional reduction of %LDL_4_ (i.e., sdLDL) only occurred in the BOLD+ intervention, not the DASH and BOLD diets, suggests that the BOLD+ diet improvement in LDL density profile was at least partially mediated by the replacement of carbohydrates with protein (mixed source, mainly lean beef), in addition to the effects of SFA reduction. Despite the difference in protein source, this finding is consistent with earlier observations that replacement of carbohydrate by protein in a low SFA diet can improve the lipoprotein profile by shifting LDL distribution to a larger average particle size in overweight and hypertensive subjects [8,35,36,37,38]. The underlying mechanism for the decrease in LDL_4_ by the BOLD+ diet is unknown but may involve alterations in ApoB metabolism. Higher VLDL ApoB secretion, lower VLDL ApoB fractional conversion rate, as well as a lower LDL ApoB fractional catabolic rate have been observed in healthy men with a predominance of sdLDL (pattern B) (25).

HOMA-IR is a validated measurement of insulin sensitivity in healthy participants with normal glucose tolerance. A value of 1.00 was considered to define healthy normal [39], while a HOMA-IR of ≥2.6 has been previously used as a threshold for classification of low insulin sensitivity [40], which is close to the median value of 2.73 in our sample. Our results are a secondary analysis of samples from the BOLD study [22]. That study did not control for baseline HOMA-IR, and baseline values calculated as a part of the secondary analysis reported for the first time herein ranged from 1.27 to 4.73. Thus, our comparisons were made on participants whose insulin sensitivity varied from near normal to markedly elevated despite having fasting glucose concentrations within normal limits. The range of values improved our ability to detect the differences in response patterns in this small study.

In this population HAD, DASH, BOLD and BOLD+ produced similar numerical reductions %LDL_4_ in the subset with baseline HOMA-IR below the median, although only the BOLD and BOLD+ diets showed statistically significant reductions relative to baseline. In contrast, %LDL_4_ responses varied significantly across diet conditions in the subset with higher baseline HOMA-IR, increasing with the HAD and DASH diets, remaining essentially unchanged with the BOLD diet, and decreased with the BOLD+ diet. The BOLD+ diet elicited a reduction in %LDL_4_ in the higher HOMA-IR subgroup numerically similar (*p* > 0.05) to that observed with DASH, BOLD or BOLD+ test diets among subjects with lower HOMA-IR. The limited effect of BOLD+ in the higher HOMA-IR may relate to their higher BMI compared to the lower HOMA-IR group (24.2 ± 0.6 vs. 26.9 ± 0.9, *p* < 0.001, Appendix A Table A2). Recent studies by some of our group demonstrated that weight loss is the primary driver of metabolic syndrome resolution [41,42].

Changes were observed in additional lipoprotein density subfractions. Consistent with other findings that decreases in large HDL_2_, i.e., HDL_2b_, are the most pronounced HDL response in healthy subjects who are shifted to diets low in total fat and SFA [43], is the reduction in HDL_2b_ following consumption of the DASH and BOLD diets compared to HAD. BOLD+ appeared to modestly reverse the significant decrease in absolute HDL_2b_ concentration induced by the DASH and BOLD dietary patterns as amounts were not different from those found on the HAD dietary pattern. Apolipoprotein measurements in the original BOLD study showed that HDL ApoC-III was significantly reduced by BOLD+ compared with HAD [22]. ApoC-III preferentially associates with large HDL_2b_ subfractions [44] and when enriched in ApoCIII, the anti-inflammatory functionality of HDL_2b_ can be compromised [45] and increase risk of cardiovascular disease [46]. A reduction in ApoC-III combined with no change in HDL_2b_ amount may suggest an improvement in HDL functionality by the BOLD+ dietary pattern.

The DASH diet emphasized inclusion of complex carbohydrates, plant and lean animal protein sources in the context of dietary pattern rich in fruit, whole grains, and vegetables that is lower in total and SFA. The results of our secondary analysis suggest that recommendations for lifestyle change to alter modifiable risk factors and reduce ischemic heart disease that include reductions in total and SFA intakes should consider increasing protein at the expense of carbohydrate while retaining a fiber intake greater than 30 g/day to achieve a BOLD+-type dietary pattern. This dietary pattern seems most appropriate for individuals with elevated HOMA-IR. The lean, unprocessed beef (153 g/day) included in the BOLD+ diet pattern provides a complete protein and is rich in iron, zinc, and the vitamins niacin, B_6_, and B_12_ [47]. These vitamins and minerals are important for antioxidant defenses and tissue repair processes [48,49] and may contribute to the results reported. Daily consumption of this quantity of beef in the context of a BOLD+ dietary pattern and weight maintenance resulted in reduced sdLDL total and fractional amounts in the absence of a reduction in HDL_2b_.

## 5. Conclusions

In summary, we found that increasing dietary protein, at the expense of carbohydrate, in a low-SFA, heart healthy diet had favorable effects on LDL particle distribution. A low SFA, moderate protein diet including lean beef (BOLD+) improved the LDL density profile by decreasing the absolute and proportional abundance of sdLDL (LDL_4_), compared to a higher-SFA HAD, in a mildly hypercholesterolemic study cohort. The results are also consistent with the hypothesis that the %LDL_4_ response to dietary modifications may differ according to baseline level of insulin resistance as assessed by HOMA-IR. Among subjects with higher baseline HOMA-IR values, the two low-SFA diets did not elicit reductions in %LDL_4_ relative to the HAD, whereas levels were reduced with the BOLD+ diet to a degree that was similar to that produced by all three of the low-SFA diets among subjects with lower HOMA-IR values.

## Figures and Tables

**Table 1:** Energy and macronutrient profiles of the BOLD study diets.

	HAD	DASH	BOLD	BOLD+
95% Lean beef (g/day)	20	28	113	153
Calories (kcal)	2097	2106	2100	2104
Protein (%E)	17	18	19	27
Carbohydrate (%E)	50	55	54	45
Fat (%E)	33	27	28	28
SFA (%E)	12	6	6	6
MUFA (%E)	11	9	11	12
PUFA (%E)	7	8	7	7

**Table 2:** Baseline characteristics of study participants.

	Men (*n* = 10)	Women (*n* = 17)	Total (*n* = 27)
Age (years)	50.9 ± 2.6	50.9 ± 2.2	50.9 ± 1.7
BMI	26.5 ± 0.8	25.1 ± 0.9	25.6 ± 0.6
TG (mmol/L)	1.08 ± 0.10	1.10 ± 0.08	1.09 ± 0.06
TC (mmol/L)	4.98 ± 0.15	5.84 ± 0.18 *	5.52 ± 0.15
LDL-C (mmol/L)	3.26 ± 0.13	3.89 ± 0.14 *	3.65 ± 0.12
HDL-C (mmol/L)	1.23 ± 0.07	1.45 ± 0.10	1.37 ± 0.07
ApoB (mg/dL)	89.0 ± 2.73	97.9 ± 1.82 *	94.5 ± 8.46
Glucose (mmol/L)	4.94 ± 0.08	4.50 ± 0.07	4.68 ± 0.05
Insulin (mU/L)	12.70 ± 1.50	13.20 ± 0.70	13.00 ± 0.70
HOMA-IR	2.79 ± 0.34	2.65 ± 0.16	2.71 ± 0.17
hsCRP (mg/L)	1.00 ± 0.16	1.09 ± 0.22	1.06 ± 0.15

Data are expressed as mean ± SEM. * *p* <0.05 vs. men. Skewed data were log transformed before statistical analysis.

**Table 3:** Effect of diets on clinical and biochemical parameters.

	HAD (*n* = 27)	DASH (*n* = 27)	BOLD (*n* = 27)	BOLD+ (*n* = 27)
BMI	24.5 ± 0.70	25.3 ± 0.60	25.5 ± 0.60	25.4 ± 0.60
TG (mmol/L)	1.02 ± 0.07	1.04 ± 0.06	1.01 ± 0.06	0.98 ± 0.06
TC (mmol/L)	5.26 ± 0.16	5.01 ± 0.16 *	5.04 ± 0.17 *	4.96 ± 0.16 *^,†^
LDL-C (mmol/L)	3.51 ± 0.12	3.30 ± 0.11 *	3.34 ± 0.13 *	3.29 ± 0.12 *
HDL-C (mmol/L)	1.28 ± 0.06	1.19 ± 0.06 *^,†^	1.22 ± 0.06 *	1.20 ± 0.06 *^,†^
ApoB (mg/dL)	95.1 ± 2.8	92.2 ± 2.8	92.0 ± 3.5	91.8 ± 2.9
Glucose (mmol/L)	4.83 ± 0.08	4.75 ± 0.07	4.83 ± 0.07	4.91 ± 0.08
Insulin (IU/mL)	12.8 ± 0.8	12.3 ± 0.7	13.6 ± 0.8	13.4 ± 0.7
HOMA-IR	2.76 ± 0.20	2.64 ± 0.14	2.93 ± 0.17	2.94 ± 0.17
hsCRP (mg/L)	1.07 ± 0.25	0.99 ± 0.19	0.92 ± 0.13	0.93 ± 0.12

Data are expressed as mean ± SEM. *n* = 25 for HOMA-IR; *n* = 24 for CRP. * *p* <0.05 vs. baseline; ^†^ *p* < 0.05 vs. HAD (Tukey HSD). Skewed data were log transformed before statistical analysis.

**Table 4:** Lipoprotein density distribution at baseline and after the test diets.

	Baseline (*n* = 27)	HAD (*n* = 27)	DASH (*n* = 27)	BOLD (*n* = 27)	BOLD+ (*n* = 27)
Lipoprotein distribution calculated as absolute AUC					
TRL	216 ± 16	204 ± 22	214 ± 14	181 ± 14	195 ± 23
LDL_1_	56 ± 5	44 ± 3	47 ± 4	45 ± 4	52 ± 5
LDL_2_	274 ± 15	240 ± 15	232 ± 14 *	226 ± 14 *	253 ± 21
LDL_3_	780 ± 49	730 ± 47	665 ± 48 *	667 ± 48 *	687 ± 38
LDL_4_	844 ± 39	822 ± 35	797 ± 21	761 ± 21	723 ± 33 *^,†^
HDL_2b_	369 ± 28	370 ± 28	337 ± 22 *^,†^	331 ± 22 *^,†^	345 ± 29
HDL_2a_	438 ± 22	422 ± 24	406 ± 20	408 ± 20	420 ± 26
HDL_3a_	422 ± 12	395 ± 12	408 ± 12	394 ± 12	399 ± 12
HDL_3b_	364 ± 9	343 ± 9	353 ± 8 *	329 ± 8 *	336 ± 9 *
HDL_3c_	128 ± 5	127 ± 5	129 ± 4	118 ± 4	124 ± 5
Lipoprotein distribution calculated as % total AUC					
TRL	5.5 ± 0.4	5.6 ± 0.6	5.9 ± 0.4	5.3 ± 0.4	5.6 ± 0.6
LDL_1_	1.5 ± 0.1	1.2 ± 0.1	1.3 ± 0.1	1.3 ± 0.1	1.5 ± 0.1
LDL_2_	7.1 ± 0.3	6.4 ± 0.3	6.4 ± 0.3	6.5 ± 0.3	7.0 ± 0.3
LDL_3_	19.8 ± 0.9	19.4 ± 0.8	18.3 ± 1.0	18.9 ± 1.0	19.1 ± 0.7
LDL_4_	21.8 ± 0.8	22.5 ± 0.8	22.4 ± 0.4	22.4 ± 0.4	20.8 ± 0.5 ^†,‡,§^
HDL_2b_	9.3 ± 0.4	9.9 ± 0.4	9.3 ± 0.3	9.4 ± 0.3	9.5 ± 0.5
HDL_2a_	11.2 ± 0.2	11.3 ± 0.2	11.3 ± 0.2	11.7 ± 0.2	11.8 ± 0.3
HDL_3a_	10.9 ± 0.2	10.7 ± 0.2	11.5 ± 0.2 *^,†^	11.5 ± 0.2 *^,†^	11.5 ± 0.3^ †^
HDL_3b_	9.5 ± 0.2	9.4 ± 0.3	9.9 ± 0.2	9.6 ± 0.2	9.7 ± 0.3
HDL_3c_	3.4 ± 0.1	3.5 ± 0.2	3.6 ± 0.1	3.5 ± 0.1	3.6 ± 0.1

Data are expressed as mean ± SEM. *n* = 27. * *p* <0.05 vs. baseline; ^†^ *p* <0.05 vs. HAD; ^‡^ *p* <0.05 vs. DASH; ^§^ *p* <0.05 vs. BOLD (Tukey HSD). Skewed data were log transformed before statistical analysis.

**Table 5:** Correlations between percent LDL_4_ change from baseline and baseline lipid, HOMA-IR, and CRP for four test diets.

	HAD		DASH		BOLD		BOLD+	
	*r*	*p*	*r*	*p*	*r*	*p*	*r*	*p*
TG	0.16	0.428	0.02	0.922	0.15	0.445	0.16	0.427
TC	0.16	0.412	0.27	0.161	0.13	0.502	0.21	0.300
LDL-C	0.12	0.536	0.22	0.271	0.24	0.222	0.25	0.205
HDL-C	0.21	0.299	0.21	0.288	0.18	0.356	0.05	0.813
HOMA-IR	0.36	0.065	0.56	0.003	0.47	0.018	0.13	0.533
hsCRP	0.35	0.093	0.23	0.282	0.32	0.125	0.18	0.395

Pearson correlation coefficients. For each intervention diet, *n* = 27 for lipid variables; *n* = 25 for HOMA-IR; *n* = 24 for CRP.

## Data Availability

The data presented in this study are available on request from the corresponding author. The data are not publicly available as no appropriate public database is available.

## Appendix A

**Table A1:** Reproducibility for intra- and inter- assays.

	Intra-Assay (*n* = 3)			Inter-Assay (*n* = 5)		
TG	Low	Medium	High	Low	Medium	High
Lipoprotein distribution calculated as absolute AUC						
TRL	18.6	13.9	14.9	19.8	11.3	10.6
LDL_1_	33.6	21.8	12.9	32.6	21.7	8.2
LDL_2_	10.1	5.7	8.6	11.3	6.8	4.1
LDL_3_	2.4	5.1	8.0	2.9	4.4	4.7
LDL_4_	6.2	9.4	8.4	5.4	5.1	7.6
HDL_2b_	1.5	4.6	6.4	0.2	1.4	2.9
HDL_2a_	3.4	4.7	6.1	2.0	1.5	0.4
HDL_3a_	3.2	4.6	4.4	1.9	1.7	1.9
HDL_3b_	4.3	3.3	1.8	3.0	3.4	6.8
HDL_3c_	13.9	10.8	9.3	14.4	9.5	13.4
Average	9.7	8.4	8.1	9.3	6.7	6.1
Lipoprotein distribution calculated as % total AUC						
TRL	10.8	8.8	22.9	9.6	8.8	19.1
LDL_1_	21.9	19.2	12.2	21.4	18.1	11.2
LDL_2_	5.9	4.3	8.0	4.4	3.4	4.1
LDL_3_	5.0	5.6	3.9	5.1	4.9	5.5
LDL_4_	4.3	4.3	5.9	3.4	3.5	4.5
HDL_2b_	3.2	1.9	7.2	2.2	1.9	5.9
HDL_2a_	8.7	4.2	3.8	7.7	5.0	5.8
HDL_3a_	6.1	2.3	4.3	5.5	3.4	3.8
HDL_3b_	4.6	2.0	7.3	4.2	0.8	4.0
HDL_3c_	11.7	8.4	9.9	9.9	8.2	6.1
Average	8.2	6.1	8.5	7.3	5.8	7.0

Coefficients of variation for intra-assay and inter-assay. Low TG = 0.63 mmol/L, medium TG = 1.15 mmol/L, high TG = 2.25 mmol/L.

**Table A2:** Subject characteristics following reclassification according to HOMA-IR median split.

	Lower HOMA-IR	Higher HOMA-IR
HOMA-IR	2.1 ± 0.1	3.4 ± 0.2 *
SEX, M:F, *n*	5:8	5:9
Age (years)	51.1 ± 2.3	50.8 ± 2.5
BMI	24.2 ± 0.6	26.9 ± 0.9 *
Triacylglycerol (mg/dL)	86.1 ± 5.9	106.2 ± 8.2
Total cholesterol (mg/dL)	219.8 ± 7.6	207.2 ± 8.5
LDL cholesterol (mg/dL)	145.3 ± 5.5	137.2 ± 7.2
HDL cholesterol (mg/dL)	57.3 ± 4.2	48.9 ± 3.3
hsCRP (mg/L)	1.1 ± 0.3	1.6 ± 0.4

Values are means ± SEM. * *p* < 0.001 vs. lower HOMA-IR group using unpaired *t* test. Values for HOMA-IR and hsCRP were log transformed for analysis.
